# Rheumatism and Gout

**Published:** 1901-04-20

**Authors:** 


### Rheumatism and Gout.

(*Continued from page* 454.)

English views of rheumatism in children are confirmed in many points by C. Lachmanski [11] from an examination of 73 cases. He finds that it is most prevalent in autumn, often commences with digestive troubles, pain in the abdomen, and remarkable pallor. There is little sweating and often no swelling or redness of the joints; the temperature soon comes down, but relapses, and above all cardiac complications are to be feared. Chorea may follow an attack, and salicylates, useful as they are for the rheumatism, have no effect on it. *Aspirin* as a substitute for salicylate of soda in various forms of rheumatism is highly praised by G. A. Hewitt,[12] who thinks it actually improves the action of the heart and does not tend to produce dyspepsia or noises in the head. Doses of from 5 to 20 grains may be given hourly or at longer intervals in a capsule or mixed with sugar as a powder. Liesau, of Bremen, Wohlgemuth, Witthauer, and others, also report well of it. Liesau [13] notes the remarkable sweating and rapid relief of pain, its harmlessness to the heart, and its antipyretic effects in various diseases as well as in rheumatism. He prefers to administer the total daily dose, one drachm or under, in divided portions during the afternoon only.

**Pneumoccal Arthritis** [14] is the subject of an interesting paper by E. T. Cave, of Bath, who has collected 31 cases. Most of these followed pneumonia, but a few occurred independently. Indeed, it is suggested that some of the severe forms of rheumatism are due to the ordinary rheumatic poison plus a secondary infection by pneumococci or other organisms. This form of arthritis is apt to attack a joint which has been previously injured, often takes an acute course with suppuration, and frequently proves fatal. The effusion into the joint may be serous or purulent, and in most instances the pneumococcus has been found in it; but sometimes either the effusion has been caused only by toxins or the cocci were already dead, for the cultures were sterile. Males are more often affected than females, and patients are usually advanced in life, or at least adults. There may be one or many joints affected, more often those of the upper extremity; some recover, and others may need opening and drainage. In the latter case the cartilages and the heads of the bones may be destroyed and the pus may burrow for some distance in the neighbourhood, if not treated in time. Pericardial, meningeal, and other lesions are frequently present, showing the widely spread infection, and are often the cause of death. Indeed, experiments on animals show that it is usually after a case of pneumonia has gone on for some days, and the patient has been partially immunised that the joints are attacked instead of a general septicœmia occurring, though too often this outlet for the poison is insufficient to save his life.

**Gonorrhœal Rheumatism.**—G. Lorimer,[15] from an analysis of 250 cases, concludes that we may have (*a*) an acute form appearing in the course of acute rheumatism if gonorrhœa is also present; (*b*) a subacute form without acid perspiration, showing rarely cardiac lesions from infective endocarditis, and stationary joint affections with perhaps suppuration or chronic joint changes or marked muscular atrophy; (*c*) and lastly an asthenic type, which may be mono-articular with hydro-arthrosis. In the first class true rheumatic symptoms appear at first and are then replaced by those of the gonorrhœal type. He emphasises the failure of salicylates to relieve the latter affection and the frequency with which the knees, heels, and the insertion of the plantar fasciæ are attacked. A mono-articular attack of this type is exceptional. The eye and the temporo-maxillary joints are rarely involved in true rheumatism and any trouble there should lead to the investigation of a possible gonorrhœa. Symptoms of early rheumatoid arthritis following a leucorrhœa in women, and which end in complete recovery, are possibly gonorrhœal.

**Classification of Joint Diseases.**—G. A. Bannatyne[16] divides them into toxic, nervous, and senile degenerations. In another point of view some are accidental complications while others are the chief manifestations of a disease.

In the diagnosis of these affections we may note that indurated patches or muscular nodes are found chiefly in muscular rheumatism—subcutaneous nodules, not only in rheumatism but in other diseases, such as gout and Maltese fever, and rather larger ones in rheumatoid arthritis; and that bursal enlargements and Heberden's nodes also occur in more than one distinct disease. Rheumatoid arthritis comprises two distinct conditions. There is an acute form in young persons with no cartilaginous or bony outgrowths but with tense or soft swelling of the joints and rapid atrophy of the muscles, chiefly on the proximal side of the joint affected, and particularly in the extensors and inter-ossei. Secondly, we find a chronic form in older persons showing bony outgrowths and wasting of the disused muscles only. Gonorrhœal rheumatism has several diagnostic marks, and it should be remembered that an attack may come on within a week or not till many months after the local infection, which may be in the eye (purulent ophthalmia) instead of in the urethra. A gleet may occur in gout which is not of a specific character, and the gouty swellings may then be mistaken for gonorrhœal rheumatism. The author takes as his type of scarlatinal rheumatism that which appears just as the rash is fading in severe attacks chiefly in adults. This often affects several joints and may lead to suppuration. He thinks it yields usually to salicylates when it is of a mild form. Malaria may lead to joint affections itself, and seems to render gout and rheumatism more intractable.

A. Stengel,[17] in discussing the treatment of rheumatism, advises a few large doses of salicylates instead of many divided ones. However, in acute forms 100 or 150 grains must be given daily for two or three days. Very great benefit arises from a ten or twenty per cent. ointment of salicylate of methyl applied to a joint and covered by oiled silk. Splints or plaster cases relieve the inflammation in a joint very rapidly, and are useful adjuncts to other treatment. Salacetol is said to appear in the urine in a quarter of an hour, and contains 71 per cent. of salicylic acid, against 86 found in the soda salt, 64 in salol, and 54 in salophen. It is of value, says Lasker,[18] as an intestinal antiseptic, and in cystitis and rheumatism. In the latter, especially if acute, the results are most satisfactory. Carl N. Brandt[19] denies that rheumatism and gout are distinct diseases, and regards them as merely groups of symptoms due to retention of the products of metabolism. In all cases he thinks there is a retention of urid acid, and when this is again freely excreted the various symptoms disappear. Hence he prefers the name uric acid toxæmia, and relies chiefly on diet and alkaline baths, by which he often obtains an excretion of uric acid two or three times as great as at the commencement of treatment. Citrophen has been praised by Pinggera[20] and other writers as a very useful remedy in rheumatism and one which has no bad effects on diseased hearts and does not cause nausea or noises in the head. Moreover, it seems to be a valuable antipyretic, pleasant to take, and efficacious in migraine and other nervous troubles. The relation of rheumatoid arthritis to true rheumatism is held uncertain by some writers. Thus C. O. Hawthorne[21] denies that they are proved to be distinct. He discusses the case of a young woman with mitral disease and a history of a few pains in the feet and one hand, but instead of the signs of articular rheumatism, she showed well-marked osseous outgrowths with deflection of the phalanges and some muscular wasting of the type common in rheumatoid arthritis. He confesses that both diseases may have been present, but is inclined to think that both groups of lesions are due to the same poison. Russell Forsbrook[22] finds that in acute rheumatoid arthritis guaiacol carbonate relieves the pain and reduces the temperature and swelling, especially in early cases. He claims that it is tasteless, and does not irritate the digestion, which he imagines is the case with creosote. Other writers, however, give the latter drug simply beaten up in milk for long periods without difficulty, and in doses increased gradually up to 30 and 60 minims three times a day. Creasote may also be painted on the painful joints and covered with protective, but here the amount should not exceed 10, or at most 15, drops at first, since it acts as a more powerful depressant when absorbed through the skin than when taken by the mouth.

[11] Glasgow Med. Journ., Feb. [12] Med. Bulletin, Nov. [13] Clinic Excerpts, p. 90, 1900. [14] Lancet, Jan. 12. [15] Quarterly Med. Jour. Nov. [16] Lancet, Feb. 23. [17] Med. News, Dec. 22. [18] Les Nouv. Remèdes, Nov. 24. [19] Lancet, Nov. 3. [20] Inaugural Thesis. [21] Clin. Journ., Jan. 9. [22] Lancet, Sept. 18.

